# Identification of Resolvin D1 and Protectin D1 as Potential Therapeutic Agents for Treating Kidney Stones

**DOI:** 10.1155/2022/4345037

**Published:** 2022-02-24

**Authors:** Bohan Wang, Jingchao Wei, Qi Huangfu, Fei Gao, Lanxin Qin, Jiao Zhong, Jiaming Wen, Zhangqun Ye, Xiaoqi Yang, Haoran Liu

**Affiliations:** ^1^Department of Urology, The Second Affiliated Hospital, School of Medicine, Zhejiang University, Hangzhou 310000, China; ^2^Durbrain Medical Laboratory, Hangzhou 310000, China; ^3^Department of Urology, The Second Affiliated Hospital of Kunming Medical University, Kunming 650000, China; ^4^Department of Urology, Tongji Hospital, Tongji Medical College, Huazhong University of Science and Technology, Wuhan 430000, China; ^5^Department of Urology, The First Affiliated Hospital of Anhui Medical University, Hefei 230000, China; ^6^Department of Urology, Stanford University School of Medicine, Stanford, 94305 California, USA

## Abstract

Intrarenal calcium oxalate (CaOx) crystals induce renal tubular epithelial cell (TEC) inflammatory and oxidative injury. This study is aimed at exploring potential therapeutic lipid components in kidney stones because lipids are involved in the development of several diseases and indicate the risk of kidney stones. Serum specimens were collected from 35 kidney stone patients and 35 normal controls. The lipid components in serum were measured, and differences were analyzed. The documented biological importance was comprehensively reviewed to identify lipids that differed significantly between the two groups to find potential agents associated with kidney stones. CaOx nephrocalcinosis mouse model was established to examine the therapeutic effects of specific lipids on CaOx deposition and CaOx-induced oxidative renal injury. Several lipids with significantly different levels were present in the serum of patients with stones and normal controls. Resolvin D1 (RvD1) (4.93-fold change, *P* < 0.001) and protectin D1 (PD1) (5.06-fold change, *P* < 0.001) were significantly decreased in the serum of patients with kidney stones, and an integrative review suggested that these factors might be associated with inflammatory responses, which is a crucial mechanism associated with stone damage. The administration of RvD1 and PD1 significantly inhibited kidney CaOx deposition and suppressed CaOx-induced renal tubular cell inflammatory injury and necrosis in a CaOx nephrocalcinosis mouse model. Furthermore, RvD1 and PD1 facilitated the expression of the oxidative indicator superoxide dismutase 2 (SOD2), inhibited NADPH oxidase 2 (NOX2) expression, and diminished intracellular reactive oxygen species (ROS) levels. This study preliminarily elucidated the role of lipids in kidney stones. The inhibitory effects of RvD1 and PD1 on oxidative damage induced by CaOx deposition provide a promising perspective for kidney stone treatment strategies.

## 1. Introduction

The prevalence of kidney stones, which is a disorder that affects the urinary system, has increased in various regions worldwide in the past decade [1]. According to their composition, kidney stones are mainly categorized as oxalate, phosphate, or uric acid stones. Uric acid and cystine stones are also common. Some insoluble or poorly soluble medications in the urine may also develop into stones [2]. Most kidney stones are formed of calcium oxalate (CaOx) and calcium phosphate (CaP). Kidney stones are free or attached to the kidney pelvis or calyces in the form of mineral clots. Specifically, specific minerals in the urine filtered by the kidneys become supersaturated, and then, the crystals continue to linger, grow, and eventually form stones. Proteins, chemical polymers, and other mineral crystals present in urine might also serve as nuclei for the formation of stones [3].

The recurrence risk of kidney stones can be as high as 50% following the first kidney colic within five years [4]. Such a high recurrence rate underscores the fact that the mechanism of kidney stone formation is not simple. Kidney stones are now increasingly recognized as a systemic disease. Risk factors associated with stone formation include diabetes, hypertension, and metabolic syndrome [5]. Particular focus is being given to the involvement of metabolic factors in the development of kidney stones [6]. Along with crystals, stones frequently contain a significant amount of organic matrix. These organic matrices are present within and around the crystals. A few studies have shown a relationship between crystals and the organic matrix in stone formation. It is worth mentioning that the matrix incorporates a variety of lipids [7]. Another noteworthy fact is that children have a considerably lower incidence of urolithiasis than adults. Several studies have revealed an association between abnormal lipid metabolism and the development of urolithiasis in children. Additionally, epidemiological studies have revealed an increased risk of developing kidney stones in individuals with dyslipidaemia [8]. These findings indicate that lipids may be involved in the formation of stones. However, the association between lipids and kidney stones, as well as the underlying mechanisms, remains elusive.

Lipids are engaged in various cellular metabolic processes as essential bioactive components, including the maintenance of normal cellular morphology, cellular energy metabolism, signal transduction, and substance transport. Mounting evidence shows that dyslipidaemia is associated with various diseases. For instance, dyslipidaemia is significantly associated with diabetes and hypertension, while diabetes and hypertension are well-established risk factors for kidney stones [9]. Given the relationship between these diseases and kidney stones, it would be intriguing to investigate the involvement of lipids in stone formation.

Lipidomics refers to the extensive investigation and integrated characterization of lipid structure and function on a spatial and temporal scale. The field of lipidomics research has grown rapidly since the advent of mass spectrometry, providing solid evidence for the function of lipids in various diseases. To date, there have been few lipidomics studies of kidney stones. Based on the practical needs and existing theoretical achievements, the present study used mass spectrometry to examine the potential lipid molecules associated with the formation of kidney stones, which may assist in elucidating the mechanism by which lipid metabolic components contribute to stone formation. We found that serum levels of resolvin D1 (RvD1) and protectin D1 (PD1) were significantly lower in patients with kidney stones than in normal controls, indicating their possible protective role in kidney stones. As anti-inflammatory and antioxidant agents, RvD1 and PD1 were reported to exert protective effects in many diseases. Bathina and Das found that RvD1 can prevent streptozotocin-induced type 1 diabetes by reducing oxidative stress and suppressing inflammation [10]. In mouse model of lipopolysaccharide-induced keratitis, RvD1 was reported to ameliorate the inflammatory process [11]. In addition, PD1 was also found to enhance resolution of inflammation in lipopolysaccharide-induced acute lung injury [12]. Duffield et al. demonstrated that resolvin D series and PD1 may play an important role in protecting against acute kidney injury [13]. The protective role of RvD1 and PD1 in kidney stones and the underlying mechanisms are worth further investigation. Attempts to identify therapeutic lipid molecules for kidney stones are aimed at bringing genuine insight and a basis for novel therapeutic approaches.

## 2. Materials and Methods

### 2.1. Ethics Statements

The study was conducted according to the guidelines of the Declaration of Helsinki and approved by The Institutional Research Ethics Committee of The Second Affiliated Hospital, School of Medicine, Zhejiang University, Hangzhou, China (#IR2021001315).

### 2.2. Patients

Sera were collected from 35 patients with kidney stones and 35 normal controls from December 01, 2020, to February 01, 2021, at The Second Affiliated Hospital, School of Medicine, Zhejiang University. Written informed forms were obtained from all participants. Patients who had a history of renal stones and underwent ureteral flexible lithotripsy at our institution were included in the stone group. Exclusion criteria included hypertension, diabetes mellitus, history of gallstones, urinary tract abnormalities, and hyperparathyroidism. The inclusion criteria for the normal group were good health as confirmed by physical examination. There were no exclusion criteria for the normal group.

### 2.3. Lipid Mediator Extraction

All lipid mediator standards were purchased from the Cayman Chemical Company (Ann Arbor, MI, USA). HLB SPE cartridges were purchased from Anbocis Technology (Beijing, China). All HPLC grade solvents were acquired from Sigma–Aldrich (St. Louis, MO, USA) or Merck & Co., Inc. (Kenilworth, NJ, USA). Aliquots of 100 *μ*L of thawed human serum were used. A mixture of deuterium-labelled internal standards was added to each sample, followed by 1.5 mL of cold methanol (MeOH). The samples were vortexed for 5 min and stored at -20°C overnight. Cold samples were centrifuged at 14,000 rpm for 5 min, and the supernatant was then transferred to a new tube. Samples were evaporated under nitrogen to volumes less than 50 *μ*L, after which 1 mL of acidified H_2_O (pH 3.5) was added to each sample before HLB SPE extraction as previously described [14]. In brief, the acidified samples were loaded onto an HLB SPE column, washed with water and hexane, and eluted with methyl formate. The methyl formate fractions were collected, dried under nitrogen, and reconstituted in 100 *μ*L of MeOH:H_2_O (2 : 3, *v*/*v*). Samples were centrifuged at 20,000 rpm, and 80 *μ*L of the supernatants was subjected to LC–MS/MS lipid mediator metabolomics analysis.

### 2.4. LC–MS/MS Lipid Mediator Metabolomics Platform

Determination of lipid mediators was achieved by LC–MS/MS using a Shimadzu LC-20ACXR (Shimadzu, Kyoto, Japan) and Sciex 5500 triple quadruple mass spectrometer (Sciex, MA, USA) with heated electrospray ionization (ESI). A Phenomenex Kinetex C18 column (100 × 2.1 mm, 2.6 *μ*m) was maintained at 40°C. Solvent A was composed of 100% H_2_O with 0.1% formic acid, and solvent B was 100% acetonitrile (ACN) with 0.1% formic acid. The flow rate was 0.3 mL/min, and the gradient was 35% B at 0 min, increased to 50% B until 4.0 min, increased to 95% B at 9.0 min, maintained at 95% B until 11.5 min, returned to 35% B at 12.0 min, and maintained at 35% B until 14.0 min. ESI-MS was operated in negative mode with a voltage of -4500 V, a temperature of 500°C, curtain gas at 30 psi, GS1 at 40 psi, and GS 2 at 40 psi. The mass spectrometry parameters for each compound were optimized, and a scheduled multiple reaction monitoring (MRM) strategy was applied. Retention time and specific MRM transitions were used to identify each lipid mediator. Relative quantitation with an appropriate internal standard was performed using the MutliQuant software (Sciex, MA, USA).

### 2.5. Establishment of the CaOx Deposition Mouse Model

All experimental procedures followed the rules of the National Institutes of Health Guide for the Care and Use of Laboratory Animals. Male C57BL/6J mice (6–8 weeks) were obtained from the Experimental Animal Research Centre of Hubei and were used for the animal experiment. The mice were randomly allocated to different experimental and control groups. To establish the CaOx deposition model, each mouse was intraperitoneally injected with glyoxylate (Gly) (75 mg/kg/d) or saline from day 4 to day 10 as previously described [15]. RvD1 and PD1 were obtained from Cayman Chemical (Ann Arbor, Michigan, USA). Mice in the intervention group were intraperitoneally injected with different concentrations (18.75, 31.25 *μ*g/kg/d; 75, 125 *μ*L) of RvD1 or (11.25, 18.75 *μ*g/kg/d; 75, 125 *μ*L) of PD1 from day 1 to day 10. Ten days later, all mice were sacrificed and underwent further laparotomy and assessment.

### 2.6. Detection of CaOx Crystal Deposition

Mouse kidney tissues were fixed with formalin, paraffin-embedded for routine sectioning, and then stained with haematoxylin-eosin (HE). Then, CaOx crystal deposition in the kidneys was determined using a polarized light optical microscope (Zeiss, Oberkochen, Germany). In addition, the kidney sections were stained using Pizzolato's method to further examine crystal deposition [16]. The percent area of crystal deposition per kidney section was quantified with the ImageJ software (National Institutes of Health, USA).

### 2.7. Periodic Acid-Schiff (PAS) Staining

Kidney tissue sections were stained with PAS to assess tubular injury, including tubular dilation, tubular atrophy, tubular cast formation, sloughing of tubular epithelial cells (TECs), or thickening of the tubular basement membrane. Ten nonoverlapping microscopic fields (×200) were randomly selected, and the percentage of cells with tubular injury among total TECs was calculated (scoring: none, 0; <25%, 1; 25–50%, 2; 51–75%, 3; and >75%, 4). The average score was calculated to represent the average injury level of the 10 fields examined.

### 2.8. Terminal Deoxynucleotidyl Transferase dUTP Nick-End Labelling (TUNEL) Fluorescence Staining

TUNEL fluorescence staining was conducted to assess kidney cell death using an In Situ Cell Death Detection Kit (Roche, Rotkreuz, Switzerland) according to the manufacturer's instructions [17]. The number of TUNEL-positive cells was counted in ten randomly chosen magnification fields for each slice.

### 2.9. Immunohistochemistry (IHC)

Mouse kidneys were fixed with formalin and embedded in paraffin for routine sectioning, followed by HE staining. For immunohistochemical staining, the slices were incubated with primary antibodies against superoxide dismutase 2 (SOD2) (1 : 800, Boster, BM4813) and NADPH oxidase 2 (NOX2) (1 : 200, Boster, BA2811) overnight at 4°C and then visualized by an Envision HRP Polymer system (Boster, Wuhan, China). Image capture (×200 magnification) was performed with an Olympus BX51 microscope (Olympus, Japan). Relative expression levels were analyzed using the ImageJ software.

### 2.10. Dihydroethidium (DHE) Fluorescence Analysis

Mouse kidneys were washed with phosphate-buffered saline and then frozen immediately. Subsequently, the frozen tissues were cut into sections (5 *μ*m) using a freezing microtome and incubated with a 5 *μ*M DHE solution at 37°C for 30 min. The slides were rinsed with phosphate-buffered saline and sealed with an antifluorescence quenching agent. They were then observed and imaged under a fluorescence microscope (Nikon, Japan). Fluorescence intensity was quantified with the ImageJ software (National Institutes of Health, USA).

### 2.11. Measurement of Blood Urea Nitrogen (BUN) and Serum Creatinine

Blood samples were collected from the mice on day 10. Serum levels of BUN and creatinine were determined using commercial kits (Stanbio Laboratory, Boerne, TX, USA).

### 2.12. Statistical Analysis

The Shapiro–Wilk test was used to determine the normality of continuous variables. The *P* value was calculated by Student's *t* test and adjusted by Bonferroni correction, and values larger than 0.05 were considered to represent statistically significant differences. Measurement results from human serum are expressed as the mean ± standard deviation (SD). To identify differential lipid mediators, a *P* value of 0.001 and a fold change (FC) greater than 1.5 were used as cut-off values. Heat maps and principal component analysis were used to visualize the characteristics of all differential lipid mediators based on the quantile-normalized values. Data analysis and plots were generated using R version 4.0.4 [18] and GraphPad Prism version 8.0.1 (GraphPad Software, CA, USA).

## 3. Results

### 3.1. Identification of Differential Lipids in Serum between Kidney Stone Patients and Healthy Controls

**Figure 1:**
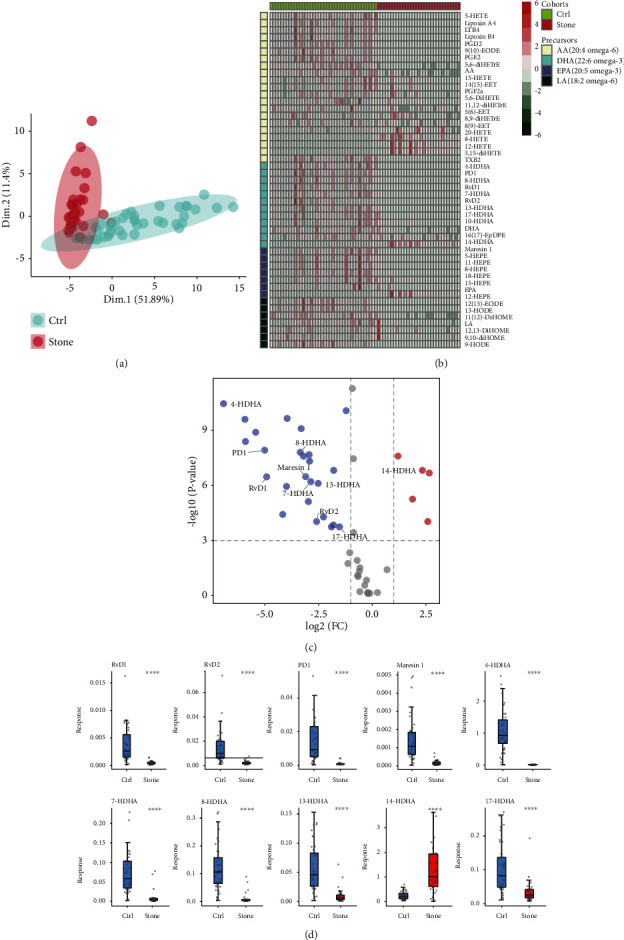
Identification of significantly reduced DHA-derived lipid mediators of serum in urolithiasis samples. (a) Principal component analysis of lipid mediators using expression data. (b) Lipid mediators are shown in a heat map. Lipid mediators are colour-coded based on the different polyunsaturated fatty acid (PUFA) precursors. (c) Volcano plots showing lipids that were differentially expressed in serum from stone patients and healthy controls. Red/blue dots represent up- or downregulated proteins, respectively, with a FC cut-off of 2 and a *P* value < 0.005. (d) Relative quantitation of DHA and DHA-derived lipid mediators in human serum. AA: arachidonic acid; DHA: docosahexaenoic acid; EPA: eicosapentaenoic acid; LA: linoleic acid.

To examine potential lipid components that may be associated with stone formation, serum specimens were collected from 35 stone patients and 35 healthy controls at The Second Affiliated Hospital, School of Medicine, Zhejiang University. There were no differences between the two groups in terms of sex ratio, age, height, or weight (Table S1). Mass spectrometry analysis of the lipid components in these blood specimens was performed. Principal component analysis based on the levels of all lipid components showed that samples from stone patients and healthy controls were clustered and were significantly different between the two groups. These findings established the reliability of the mass spectrometry results and demonstrated that the sera of stone patients and healthy controls contained distinct lipids (Figure 1(a)). Hierarchical clustering with heat map classification based on lipid precursors and serum lipid levels directly revealed the lipids that differed between the serum of kidney stone patients and healthy controls. Notably, multiple products of docosahexaenoic acid (DHA) consistently showed “elimination” in the serum of patients with kidney stones (Figure 1(b)). These results revealed some serum lipids that may be associated with kidney stone formation.

### 3.2. Verification of Serum Lipid Levels in Patients with Kidney Stones

A “volcano plot” was drawn according to the differences (∣Log_2_FC | ≥1; *P* < 0.05) in DHA products between the two groups to visualize the lipids that may be associated with kidney stones (Figure 1(c)). To further confirm the levels of these lipids in kidney stone patients, serum was collected again from the aforementioned 35 kidney stone patients and 35 healthy controls. DHA products in the serum were measured. The results were consistent with the mass spectrometry results. Specifically, 14-HDHA was significantly higher in patients with kidney stones than in healthy controls, whereas 17-HDHA, 13-HDHA, RvD2, RvD1, 4-HDHA, 8-HDHA, PD1, Maresin 1, and 7-HDHA were significantly lower in the serum of patients with kidney stones than in healthy controls (Figure 1(d)). These results suggest an association between DHA products and kidney stone formation.

### 3.3. RvD1 and PD1 Attenuated CaOx Deposition and Cellular Damage in the Kidneys

**Figure 2:**
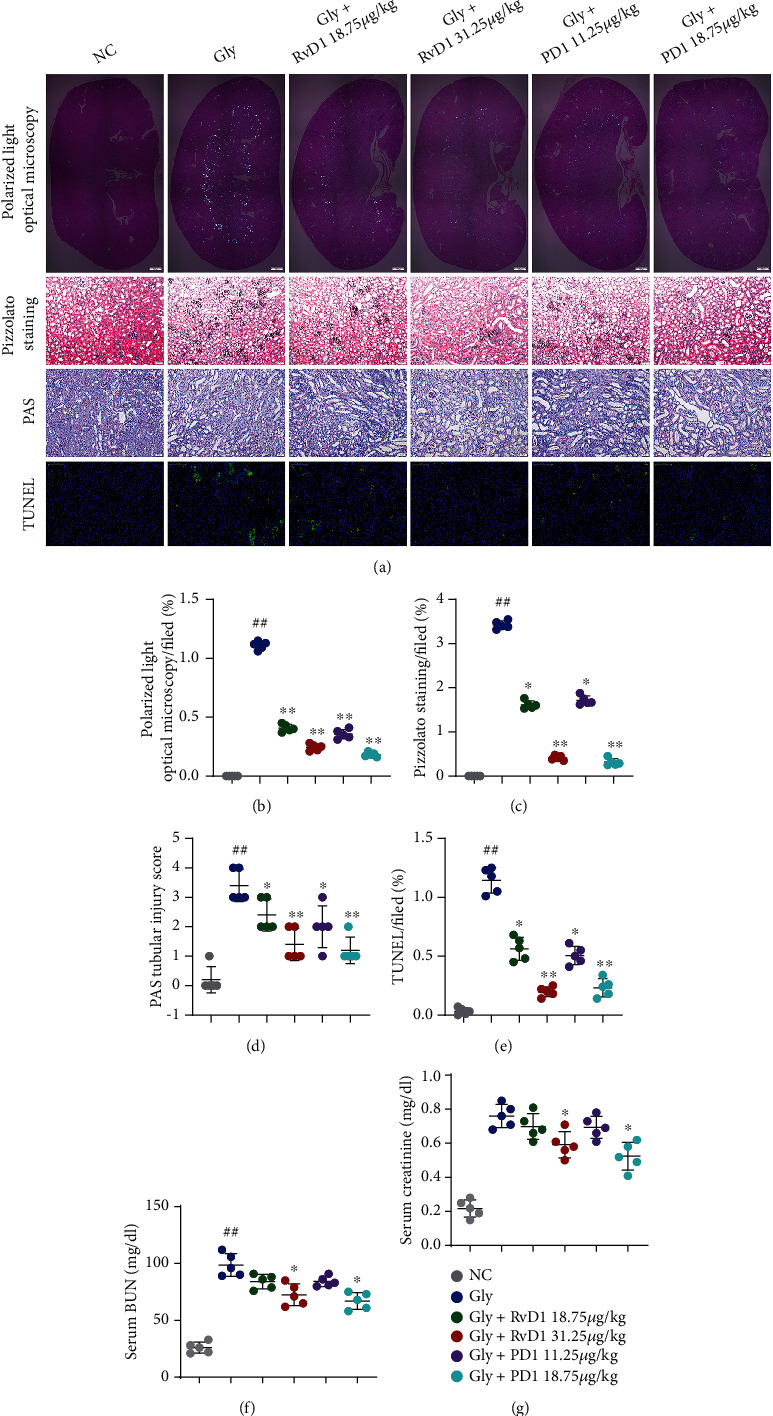
RvD1 and PD1 inhibited CaOx deposition-induced cell damage. A mouse model of CaOx deposition was constructed, and the effects of RvD1 and PD1 on CaOx deposition were observed after the administration of different concentrations of RvD1 (18.75 and 31.25 *μ*g/kg) and PD1 (11.25 and 18.75 *μ*g/kg). (a) Representative map of CaOx deposition in the whole kidney as observed by polarized light optical microscopy (×20, scale bar: 500 *μ*m). Pizzolato staining was performed to examine CaOx deposition in local tissues (×200; scale bar: 20 *μ*m). PAS staining was performed to examine cell damage (×200; scale bar: 20 *μ*m). A TUNEL assay was performed to examine apoptosis (×200; scale bar: 100 *μ*m). (b) The ratio of the areas of kidneys with crystal deposition, as determined by polarized light optical microscopy. (c) The ratio of the areas of kidneys with corticomedullary junction area crystal deposition. (d) The tubular injury score was determined by PAS staining. (e) The average number of TUNEL-positive cells per high-power field. (f, g) BUN and serum creatinine were used to assess renal function (*n* = 5). The data are the means ± SEM of three independent experiments. One representative plot of *n* = 5 mice is shown. #*P* < 0.05; ##*P* < 0.01 compared with NC, ^∗^*P* < 0.05; ^∗∗^*P* < 0.01 compared with Gly, one-way ANOVA (b–g).

RvD1 and PD1 are known to have stronger biological functional activity than other members of the DHA family and can block proinflammatory neutrophil and macrophage migration, which plays a key role in inflammatory renal injury. To investigate the therapeutic effects of RvD1 and PD1 on CaOx nephrocalcinosis, we pretreated mice with RvD1 and PD1 for 3 days and developed a CaOx nephrocalcinosis mouse model by intraperitoneal injection of Gly. Polarized light optical microscopy and Pizzolato staining showed that Gly significantly induced CaOx crystal deposition in the kidneys of mice, while RvD1 and PD1 reversed this effect in a dose-dependent manner. PAS and TUNEL staining further confirmed that RvD1 and PD1 reduced CaOx crystal-induced kidney damage (Figures 2(a)–2(e)). In addition, we measured the levels of serum creatinine and BUN to evaluate kidney function and tubular injury. We observed gradual recovery of kidney function during the treatment with RvD1 and PD1 in a dose-dependent manner (Figures 2(f) and 2(g)).

### 3.4. RvD1 and PD1 Protected against CaOx Deposition by Inhibiting Oxidative Damage in the Kidneys

**Figure 3:**
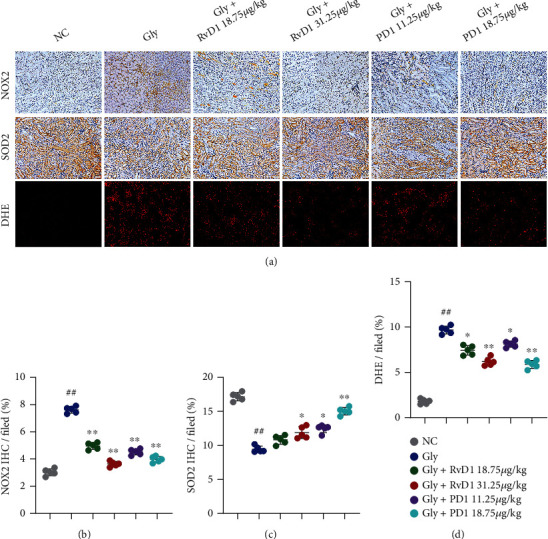
RvD1 and PD1 reduced kidney injury by inhibiting oxidative damage. (a) Treatment with different concentrations of RvD1 (18.75 and 31.25 *μ*g/kg) and PD1 (11.25 and 18.75 *μ*g/kg) in the CaOx deposition mouse model. Representative plots showing NOX2 and SOD2 are presented (×200; scale bar: 20 *μ*m). Intracellular ROS levels were measured by the DHE method (×200; scale bar: 20 *μ*m). (b–d) The ratio of the areas with positive expression of NOX2, SOD2, and DHE, as determined by IHC. One representative plot of *n* = 5 mice is shown. #*P* < 0.05; ##*P* < 0.01 compared with NC, ^∗^*P* < 0.05; ^∗∗^*P* < 0.01 compared with Gly, as determined by one-way ANOVA (b–d).

**Figure 4:**
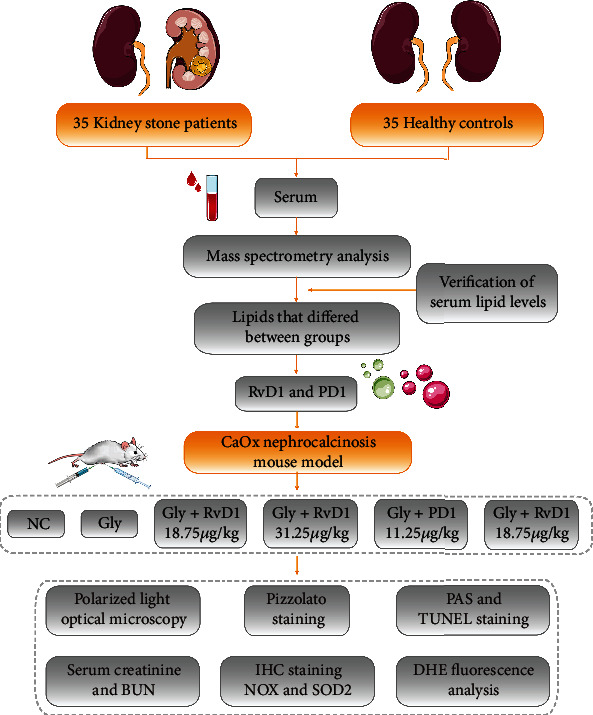
A flow chart of study design in human samples and mouse model.

To further explore the mechanism by which RvD1 and PD1 inhibit CaOx-induced kidney inflammation and oxidative damage and thereby reduce CaOx crystal deposition, we examined reactive oxygen species- (ROS-) related indicators. NOX2 is a superoxide-generating enzyme that forms ROS [19]. SOD2 degrades superoxide, a toxic byproduct of the mitochondrial electron transport chain, to form hydrogen peroxide and diatomic oxygen. This function enables SOD2 to eliminate mitochondrial ROS and thereby protect cells from death [20]. Immunohistochemical staining of NOX2 and SOD2 showed that RvD1 and PD1 reversed the effects of Gly-induced oxidative damage (Figures 3(a)–3(c)). Furthermore, we measured intracellular ROS using the DHE approach. Both RvD1 and PD1 significantly reduced ROS levels (Figures 3(a) and 3(d)). Collectively, these data suggested that RvD1 and PD1 inhibited CaOx deposition and antagonized the risk of kidney stones by inhibiting ROS (Figure 4).

## 4. Discussion

Kidney stones pose a significant risk to human health and impose a substantial economic burden on society. Urinary stone formation is a complex process involving multiple factors. The mechanisms of stone formation involve CaOx crystallization, crystal growth, aggregation, and adhesion to renal tubular epithelial cells have been extensively studied during the last few decades [21]. Although numerous investigations on the formation of kidney stones have been conducted and several theories have been established, the mechanisms of kidney stone formation are not yet unequivocally understood. A few pieces of evidence have shown that lipid components might play a role in the formation of kidney stones [22]. For instance, oxidized low-density lipoprotein (LDL) is abundant in patients with kidney stones. In addition, glycolipids and cholesterol are involved in the formation of kidney stones [23]. However, a limited number of papers are available about kidney stones and lipidomics. In this study, serum lipids were measured, and patients with kidney stones had considerably lower DHA metabolites than healthy individuals. Further exploration revealed that the DHA products RvD1 and PD1 reduced CaOx deposition in the kidneys. In addition, RvD1 and PD1 alleviated kidney inflammation and oxidative damage, which might be the mechanism by which RvD1 and PD1 reduced CaOx deposition. As CaOx deposition is a critical process in the formation of CaOx stones, RvD1 and PD1 might protect against the development of kidney stones.

Both RvD1 and PD1 are produced from DHA, a long-chain, highly unsaturated omega-3 (n-3) fatty acid that is one of the main components of fish oils. DHA is involved in the regulation of various cellular activities and functions, including altering membrane structure, membrane protein function, cell signalling, and lipid mediator production [24]. Endogenous conversion of DHA into resolvins, protectins, and maresins has been demonstrated. DHA is currently broadly used as a dietary supplement, and a considerable body of evidence indicates that DHA has numerous health benefits. DHA significantly improved oxidative stress in kidneys of LPS-challenged mice by restoring oxidation and antioxidant balance [25]. EPA and DHA seem to help lag the progression of chronic kidney disease by decreasing the production of ROS and lipid peroxidation, promoting mitochondrial damage and tissue inflammation, which result in glomerular and tubular lesions [26]. However, the molecular mechanisms remain unclear [27].

Resolvins and protectins dominate the resolution phase of inflammation. Protectins are inflammatory mediators that inhibit leukocyte activation [28]. These factors foster the natural resolution of acute inflammation by diminishing proinflammatory mediators and clearing inflammatory cells. PD1 belongs to the resolvin family. PD1 is present in various tissues, including the retina, lung, and nervous system. PD1 has anti-inflammatory, antiapoptotic, and neuroprotective effects [29]. PD1 protects cells by reducing oxidative stress-induced inflammation and inhibiting apoptotic signalling pathways. Resolvins are lipid mediators derived from eicosapentaenoic acid (EPA) and DHA. RvD1 is physiologically produced by the sequential oxygenation of DHA by 15- and 5-lipoxygenases. RvD1 inhibits polymorphonuclear leukocyte transendothelial migration, which is an early event in acute inflammation. In addition to being anti-inflammatory, RvD1 promotes the resolution of inflammation [30]. This study provides new evidence that RvD1 and PD1 are anti-inflammatory and antioxidant agents.

Inflammation, oxidant-antioxidant imbalance, and urea cycle disruption have been established as plausible mechanisms underlying stone formation [31]. Different metabolic lipids are involved in inflammatory and oxidative stress responses, which is consistent with the kidney stone hypothesis, such as the calcium plaque theory and the supersaturated crystalline theory. Inflammation is a multifaceted and dynamic process and is mainly mediated by inflammatory factors. The inflammatory response is pivotal in homeostasis and disease development in the body. Numerous hard-to-treat disorders, including cardiovascular disease, diabetes, and neurological issues, have been intimately connected with uncontrolled inflammation [32]. Emerging evidence suggests the pivotal role of specific lipid mediators in modulating inflammation resolution [33]. The present study showed that the DHA products RvD1 and PD1 reduce CaOx deposition by alleviating the ROS-induced inflammatory response, providing strong evidence for the inhibitory effect of active lipid components on inflammation and expanding our understanding of the role of lipid components in inflammation regulation.

ROS are critical regulators of cell signalling. Low concentrations of ROS facilitate cellular adaptability to changing microenvironments through a variety of approaches. Consequently, ROS promote apoptosis and thus assist in eliminating severely damaged cells. However, high concentrations of ROS contribute to the development of various diseases, including kidney stones. Increased prooxidant damage indicators have been observed in the urine of patients with kidney stones [34]. There are multiple associations between ROS and kidney stone formation, and an imbalance between prooxidants and antioxidants drives kidney stone formation [35]. Lipids such as polyunsaturated fatty acids, which are peroxidized, can cause damage to cells, affecting protein activity and the physical properties of cell membranes. There is evidence that these oxidized lipids damage the kidney tubules and significantly contribute to the generation of kidney stones. Interestingly, it has been found that the deposition of CaOx crystals causes the activation of NOX through a complex mechanism, which in turn exacerbates the production of ROS [36]. The current study revealed that various lipid components might harm or protect the kidney. RvD1 and PD1 inhibited the deposition of CaOx through a mechanism that inhibited oxidative damage and thus suppressed the formation of kidney stones.

The regulatory mechanisms of SOD2 and NOX2 by RvD1 and PD1 remain unclear. There is evidence showing that RvD1 inhibits NOX activity by reducing the release of IL-1*β* and dephosphorylating specific proteins [37, 38]. Furthermore, RvD1-mediated inhibition of NOX was dependent on cAMP-activated protein kinase (PKA) signaling [38]. The inhibition of ROS-mediated damage by PD1 is mediated by dephosphorylation of B-cell lymphoma-extra large and is dependent on activation of phosphoinositide 3-kinase/protein kinase B signaling [39–41]. Nevertheless, the regulatory mechanisms of RvD1 and PD1 on SOD2 and NOX2 still need more exploration.

There are some limitations in this study. The specific mechanisms underlying the reduced levels of RvD1 and PD1 in patients with kidney stones were not investigated in depth, and further elucidation of this issue will be necessary. In addition, we bear in mind the difference between animal experiments and humans, so whether the conclusions drawn from the animal experiments in this study are applicable to humans is also subject to further study.

## 5. Conclusions

Although investigations targeting kidney stones are still in their early stages, there is a lack of significant, evidence-based insight into the role of lipids. Our findings shed light on the lipid changes associated with kidney stone formation. The inhibitory effect of RvD1 and PD1 on CaOx deposition suggests therapeutic strategies for kidney stones. These findings establish a preliminary theoretical basis for the development of innovative kidney stone therapeutic strategies.

## Data Availability

Data are included within the article or can be obtained from the authors upon request. The supplementary material and raw data for this article can be found online at https://www.jianguoyun.com/p/DbDYz7IQlM6BChj17J8E.

## Abbreviations

CaOx: Calcium oxalate
RvD1: Resolvin D1
PD1: Protectin D1
SOD2: Superoxide dismutase 2
NOX2: NADPH oxidase 2
ROS: Reactive oxygen species
CaP: Calcium phosphate
MeOH: Methanol
ESI: Electrospray ionization
ACN: Acetonitrile
MRM: Multiple reaction monitoring
Gly: Glyoxylate
HE: Haematoxylin-eosin
PAS: Periodic acid-Schiff
TECs: Tubular epithelial cells
TUNEL: Terminal deoxynucleotidyl transferase dUTP nick-end labelling
IHC: Immunohistochemistry
DHE: Dihydroethidium
BUN: Blood urea nitrogen
SD: Standard deviation
FC: Fold change
DHA: Docosahexaenoic acid
LDL: Low-density lipoprotein
EPA: Eicosapentaenoic acid.

## Supplementary Materials

Supplementary Materials Supplementary 1. Supplementary Table S1: baseline characteristics.
