# Correction to “Identification of Resolvin D1 and Protectin D1 as Potential Therapeutic Agents for Treating Kidney Stones”

**DOI:** 10.1155/omcl/9857091

**Published:** 2026-04-10

B. Wang, J. Wei, Q. Huangfu, F. Gao, L. Qin, J. Zhong, J. Wen, Z. Ye, X. Yang, and H. Liu, “Identification of Resolvin D1 and Protectin D1 as Potential Therapeutic Agents for Treating Kidney Stones,” *Oxidative Medicine and Cellular Longevity*, 2022, https://doi.org/10.1155/2022/4345037.

In the article, the authors have identified an error in which the incorrect number of participants was stated throughout the article. The correct number of participants is 40 controls and 31 kidney stone patients. Figure 4 is therefore updated to reflect this and is shown below:

**Figure 4:**
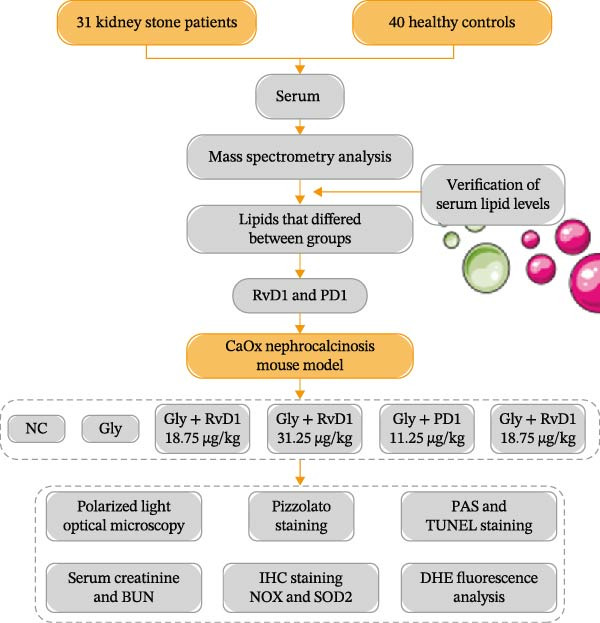
A flow chart of study design in human samples and mouse model.

We apologize for this error.

