# Supplementary material for: Identification of Resolvin D1 and Protectin D1 as Potential Therapeutic Agents for Treating Kidney Stones
Source: Oxid Med Cell Longev. 2022 Feb 24;2022:4345037. doi: 10.1155/2022/4345037 (PMC8894018; doi:10.1155/2022/4345037)
Supplement: Supplementary Materials — Supplementary 1. Supplementary Table S1: baseline characteristics. [file 4345037.f1.docx]

Table S1. Baseline characteristics

| Characteristic | Kidney stones | Normal control | P |
| --- | --- | --- | --- |
| n | 35 | 35 |  |
| Gender, n (%) |  |  | 0.811 |
| Female | 16 (22.9%) | 18 (25.7%) |  |
| Male | 19 (27.1%) | 17 (24.3%) |  |
| Age, mean ± SD | 54.63 ± 11.83 | 57.89 ± 11.75 | 0.252 |
| Height, mean ± SD | 163.77 ± 8.78 | 166.61 ± 8.69 | 0.178 |
| Weight, mean ± SD | 65.11 ± 11.88 | 68.53 ± 8.64 | 0.173 |
